# Gluten-free diet may alleviate depressive and behavioural symptoms in adolescents with coeliac disease: a prospective follow-up case-series study

**DOI:** 10.1186/1471-244X-5-14

**Published:** 2005-03-17

**Authors:** Päivi A Pynnönen, Erkki T Isometsä, Matti A Verkasalo, Seppo A Kähkönen, Ilkka Sipilä, Erkki Savilahti, Veikko A Aalberg

**Affiliations:** 1 Hospital for Children and Adolescents, Helsinki University Central Hospital, Helsinki, Finland; 2 Department of Mental Health and Alcohol Research, National Public Health Institute, Helsinki, Finland; 3 BioMag Laboratory, Engineering Center, Helsinki University Central Hospital, Cognitive Brain Research Unit, University of Helsinki, Helsinki, Finland

## Abstract

**Background:**

Coeliac disease in adolescents has been associated with an increased prevalence of depressive and disruptive behavioural disorders, particularly in the phase before diet treatment. We studied the possible effects of a gluten-free diet on psychiatric symptoms, on hormonal status (prolactin, thyroidal function) and on large neutral amino acid serum concentrations in adolescents with coeliac disease commencing a gluten-free diet.

**Methods:**

Nine adolescents with celiac disease, aged 12 to 16 years, were assessed using the semi-structured K-SADS-Present and Lifetime Diagnostic interview and several symptom scales. Seven of them were followed at 1 to 2, 3, and 6 months on a gluten-free diet.

**Results:**

Adolescent coeliac disease patients with depression had significantly lower pre-diet tryptophan/ competing amino-acid (CAA) ratios and free tryptophan concentrations, and significantly higher biopsy morning prolactin levels compared to those without depression. A significant decrease in psychiatric symptoms was found at 3 months on a gluten-free diet compared to patients' baseline condition, coinciding with significantly decreased coeliac disease activity and prolactin levels and with a significant increase in serum concentrations of CAAs.

**Conclusion:**

Although our results of the amino acid analysis and prolactin levels in adolescents are only preliminary, they give support to previous findings on patients with coeliac disease, suggesting that serotonergic dysfunction due to impaired availability of tryptophan may play a role in vulnerability to depressive and behavioural disorders also among adolescents with untreated coeliac disease.

## Background

Coeliac disease is an under-diagnosed autoimmune type of gastrointestinal disorder resulting from gluten ingestion in genetically susceptible individuals. Non-specific symptoms such as fatigue and dyspepsia are common, but the disease may also be clinically silent. Diagnosis is based on small-bowel biopsy, and a permanent gluten-free diet is the essential treatment. Undetected or neglected, coeliac disease is associated with serious complications. [1-3] Depressive symptoms [4,5] and disorders [6] are common among adult patients with coeliac disease, and depressive and disruptive behavioural disorders are highly common also among adolescents, particularly in the phase before diet treatment [7]. Recently 73% of patients with untreated coeliac disease – but only 7% of patients adhering to a gluten-free diet – were reported to have cerebral blood flow abnormalities similar to those among patients with depressive disorders [8].

Improvement in state anxiety [5], in behavioural symptoms [9], and in depressive disorders [6,10] may occur after the start of a standard gluten-free diet, and after a vitamin B-6-supplemented gluten-free diet [11]. In some cases, however, the more serious depressive episodes have appeared following the commencement of a gluten-free diet [6]. Mechanisms involved have remained unclear. Some studies have suggested the possibility of impaired availability of tryptophan and disturbances in central serotonergic function as playing a role [9,12]. In parallel with this, a significant increase in major serotonin and dopamine metabolite concentrations in the brain has been reported after one year on a gluten-free diet [13].

The present work is a preliminary prospective psychiatric follow-up study of adolescents with newly diagnosed coeliac disease measuring psychiatric symptoms, hormonal status (prolactin, thyroidal function), and large neutral amino acid (LNAA) serum concentrations repeatedly after their commencement of a gluten-free diet, testing the hypothesis that the treatment of coeliac disease may increase the availability of tryptophan and alleviate psychiatric symptoms.

## Methods

### Subjects

The study sample comprised all nine adolescents (5 girls, 4 boys; aged 14.6 ± 0.8) consecutively diagnosed with coeliac disease between January 1999 and December 2000 in the Department of the Gastrointestinal Services of the Hospital for Children and Adolescents, Helsinki University Central Hospital, in Finland. None of the patients had a history of, or current psychiatric treatment. Duration of coeliac disease symptoms and signs (abdominal pain, diarrhoea, anaemia) leading to a biopsy was 2.3 (± 1.5) years. The study was approved by the institutional Ethics Committee. Written informed consent was obtained from each patient and a parent.

### Evaluation

Baseline psychiatric evaluation was conducted 1 to 4 weeks after the diagnostic biopsy, during the wait for the diagnosis of coeliac disease to be established by the pathologist. The adolescent and a parent were interviewed separately by an adolescent psychiatrist (PP) using a semi-structured diagnostic interview, the Schedule for Affective Disorders and Schizophrenia for School-Age Children – Present and Lifetime version (K-SADS-PL) [14]. Seven patients attended the follow-up visits with laboratory tests at > 1 to ≤ 2, 3, and 6 months after starting a gluten-free diet (Table 1). Baseline and follow-up behavioural problems were assessed with the Youth Self Report (YSR) [15] and the Child Behavior Checklist (CBCL) [16], completed by a parent (Table 1), and depressive and anxiety symptoms by the 21-item versions of the Beck Depression Inventory (BDI) and Beck Anxiety Inventory (BAI), the 17-item version of the Hamilton Depression Rating Scale (HAM-D), and the 14-item Hamilton Anxiety Rating Scale (HAM-A). CGAS (Children's Global Assessment) served as a part of the K-SADS-PL.

**Table 1:** Psychiatric symptoms and disease activity among adolescent CD patients (n = 7, mean ± SD)

	0 (baseline)	1–2 months	p-value^1)^	3 months	p-value^1)^	6 months	p-value^2)^
CGAS ^3)^	74.4 (± 16.5)^4)^			86.9 (± 5.6)	0.043	88.3 (± 9.2)	0.006
HAM-D^5)^	5.7 (± 7.3)			0.3 (± 0.8)	0.043	1.0 (± 1.3)	0.009
HAM-A^6)^	6.9 (± 6.9)			0.1 (± 0.4)	0.043	0.7 (± 1.3)	0.010
BDI^7)^	3.4 (± 6.5)	0.0 (± 0.0) ^8)^	n.s.^9)^	0.1 (± 0.4)	0.041	0.6 (± 1.1)	0.014
BAI^10)^	3.0 (± 2.9)	1.3 (± 1.5) ^8)^	n.s.	0.7 (± 1.1)	0.042	1.4 (± 2.2)	n.s.
CBCL^11) ^Total problems	28.3 (± 12.8)	18.4 (± 6.9)	0.028	14.0 (± 9.5)	0.028	18.4 (± 16.2)	0.007
Anxious/depressed	3.9 (± 3.1)	1.4 (± 1.7)	0.046	1.3 (± 1.5)	0.026	1.3 (± 1.1)	0.033
Aggressive behaviour	7.3 (± 4.2)	5.6 (± 2.2)	n.s.	3.6 (± 3.1)	0.039	6.0 (± 6.4)	0.047
YSR^12) ^Total problems	24.4 (± 13.8)	17.0 (± 12.5)	n.s.	8.0 (± 7.8)	0.018	9.4 (± 7.4)	0.001
Anxious/depressed	2.7 (± 3.4)	1.6 (± 2.2)	n.s.	0.3 (± 0.5)	0.026	0.6 (± 1.0)	0.002
Aggressive behaviour	5.1 (± 2.7)	4.1 (± 2.7)	n.s.	2.0 (± 2.2)	0.044	2.4 (± 1.4)	0.021
Somatic complains	4.9 (± 2.4)	3.1 (± 2.3)	0.038	2.1 (± 1.2)	0.026	2.3 (± 2.1)	0.003
S-EndoAbA^8)^	800(200–1600)	200 (5–400)	0.018	5 (5–1600)	0.027	5 (5–100)	0.001
S-tTGAbA	139 (± 158)	30 (± 35)	0.018	19 (± 26)	0.018	7 (± 8)	< 0.001
Prolactin (mU/l)^13)^	1569 (± 767)	218 (± 60)	0.028	284 (± 170)	0.018	205 (± 92)^14)^	0.019
S-T4/TSH (nmol/l:mU/l)	34 (± 6)	64 (± 38)^14)^	0.043	84 (± 53)	0.043	74 (± 32)^15)^	0.039

^1) ^non-parametric Wilcoxon signed-ranks test; compared with baseline; ^2) ^repeated measures non-parametric Friedman test; ^3) ^Children's Global Assessment; ^4) ^mean ± SD; ^5) ^Hamilton Depression Rating Scale; ^6) ^Hamilton Anxiety Rating Scale ; ^7) ^Beck Depression Inventory; ^8) ^median (min-max); ^9) ^non significant; ^10) ^Beck Anxiety Inventory; ^11) ^Child Behavior Checklist; ^12) ^Youth Self Report; ^13) ^normal: females 50–300, males 50–500; ^14) ^n = 6 ; ^15) ^n = 4

Coeliac disease activity was followed by determining serum tissue transglutaminase (S-tTGAbA) and endomysium (S-EndoAbA) autoantibodies [17]. Pre-diet blood samples for analysis of amino acids, prolactin, thyroid function [thyroxine (S-T4), thyroid-stimulating hormone (TSH)], vitamins B6 and B12, S-tTGAbA, and S-EndoAbA were obtained on the biopsy morning, and subsequent ones as a part of follow-up visits, both after overnight fasting, between 8 and 10 a.m. All nine patients had amino acid concentrations measured at baseline, and five of them during follow-up (1–2 times). A blood sample (2 ml) was drawn from the ulnar vein into a vacuum tube for serum total and free L-tryptophan, and for other large neutral amino acids (LNAA). The tube was cooled immediately and stored refrigerated in ice until centrifuged. After centrifugation, the serum was frozen and stored at -20°C for 4 to 14 months (median 7.5) until its assay for the amino acids by a modified procedure described by Qureshi et al. [18]. All the samples were analysed in a single run, in Kuopio, Finland, and free and total L-tryptophan and other LNAA's were assessed as described by Tiihonen et al. [19].

### Statistical methods

Statistical analysis was carried out with parametric and non-parametric tests as appropriate; tests for two-independent groups (T-test, Mann-Whitney U-test), for repeated measures of two-related groups (Wilcoxon signed-ranks test), for three-related groups (Friedman test) and Spearman's rank correlation testing were used. P-values (2-tailed) < 0.05 were regarded as significant.

## Results

### Baseline evaluation

At baseline, three adolescents (3/9; 33%) had a depressive disorder: two girls had major depressive disorder (MDD), one with a learning disorder not otherwise specified (NOS), and another with comorbid conduct disorder; one girl had the depressive disorder NOS. Further, one boy had a phobic disorder plus attention-deficit hyperactivity disorder, and another conduct disorder NOS. Four adolescents (44%) had no diagnosis.

Pre-diet free L-tryptophan was positively correlated with pre-diet levels of tTGAbA (n = 8; r = 0.78, P= 0.022), and negatively with vitamin B-6 (r = 0.73, P = 0.039) and S-T4 (r = 0.74, P = 0.035). Prolactin levels (Table 1) from the biopsy morning showed a positive correlation with BDI score (self-report depression inventory; r = 0.89, P = 0.001), and a negative correlation with the ratio of L-tryptophan to amino acids competing for the same cerebral uptake mechanism (CAA) (r = 0.68, P = 0.042), but not with free L-tryptophan levels. The sum of branched-chain amino acids (BCAA: valine, leucine, and isoleucine) showed no correlation with L-tryptophan or free L-tryptophan levels.

Depressive patients (n = 3/9) had significantly higher pre-diet prolactin levels (mU/l: mean ± S.D. = 2450 ± 676 vs. 1194 ± 598, Mann-Whitney U-test, P = 0.039) and pre-diet S-T4 levels (nmol/l: mean 102 ± 3.1 vs. 84 ± 16.1, Mann-Whitney U-test, P = 0.024), and significantly lower L-tryptophan/CAA ratios (100 × pmol/μl: pmol/μl: mean 10.0 ± 0.2 vs. 11.5 ± 1.7, Mann-Whitney U-test, P = 0.020) and free L-tryptophan concentrations (pmol/μl: mean 4.7 ± 0.5 vs. 8.4 ± 3.0, two-independent samples T-test, P = 0.029). Pre-diet free L-tryptophan correlated negatively with biopsy morning S-T4 level (r = -0.74, P = 0.035). No significant differences appeared in L-tryptophan (36.3 ± 5.1 vs. 43.3 ± 6.1) or in L-tyrosine concentrations, nor in BCAA and CAA levels.

### Follow-up

Two adolescents with conduct disorders, one a girl with concomitant MDD, did not adhere to the gluten-free diet and dropped out of the psychiatric follow-up. Among others (n = 7), a significant decrease in most of the problem and symptom scores of YSR and CBCL, and in BDI, BAI, and Hamilton scales was evident after 3 months on the gluten-free diet, compared to baseline (Table 1).

Celiac disease-associated antibody titres had decreased in all by the first month on a gluten-free diet, and had already normalised (= S-EndoAbA titre < 5 and S-tTGAbA titre < 8) in 4 of 7 patients by 6 months. Boys had lower biopsy morning prolactin levels (mU/l; mean 972, SD 450 vs. girls mean 2126 ± 756; one-way Anova P = 0.032), but higher levels after one month on the diet. In the first month, the S-T4/TSH ratio (nmol/l:mU/l) reflecting thyroid function increased significantly (Table 1).

An initial increase in CAAs, also in tyrosine levels, and in total and free L-tryptophan was reaching significance after one month on a gluten-free diet. By 3 months, the increases in tyrosine alone and in CAAs as a group were significant, and the increase in free L-tryptophan was approaching significance (repeated measures Friedman test n = 4, Chi-Square 6,000, df 2, P = 0.050). (Table 2)

**Table 2:** Follow-up of the patients (n = 5): psychiatric symptoms, CD activity, and amino acidconcentrations [median (min-max)].

	0 (baseline)	≥1<1.5 months^1)^	p-value^2)^	≥3 months	p-value^2)^
CBCL Total problems	34 (9–39)	21 (8–29)	**	21 (2–22)	**
YSR Total problems	20 (9–36)	11 (5–41)	n.s.	2 (0–22)	**
S-tTGAbA	42 (10–310)	5 (3–51)	**	3 (1–17)	**
Prolactin^3)^	1100 (635–2850)	256 (127–282)	n.s *	244 (110–565)	**
L-tyrosine^4)^	33 (26–44)	39 (37–70)	n.s *	40 (38–47)	**
CAA^5)^	353 (316–441)	401 (368–657)	n.s *	395 (367–568)	**
L-tryptophan^4)^	40 (32–51)	52 (45–66)	n.s *	46 (36–59)	n.s.
Tryptophan/CAA^6)^	11.3 (10.1–14.9)	12.0 (10.1–14.1)	n.s.	10.1 (9.5–12.5)	n.s.
Free L-tryptophan ^4)^	4.9 (4.5–11.8)	8.4 (5.3–10.9)	n.s *	10.6 (5.0–19.0)	n.s. * ^7)^
Free tryptophan/CAA^6)^	1.4 (1.0–3.5)	1.9 (1.0–3.0)	n.s.	2.6 (1.3–3.4)	n.s.

^1) ^amino acid concentrations; n = 4; ^2) ^Wilcoxon signed – ranks test: compared with baseline; ** = *P *< 0.05; * = P ≥ 0.05 < 0.07; ^3) ^mU/l; normal: females 50–300, males 50–500; ^4) ^pmol/μl; ^5) ^L-valine, L-leucine, L-isoleucine, L-phenylalanine, L-tyrosine; pmol/μl; ^6) ^100 × pmol/μl: pmol/μl; ^7) ^repeated Measures Friedman test, n = 4, P = 0.050

## Discussion

We observed that the majority of adolescents with coeliac disease had depressive and behavioural symptoms before their diagnosis, and that coeliac disease patients with depression (all girls) had significantly lower pre-diet tryptophan/CAA ratios and free tryptophan concentrations and significantly higher biopsy morning prolactin levels. Adolescents with coeliac disease showed improvement in psychiatric symptoms after starting a gluten-free diet, and this improvement coincided with a significant decrease in coeliac disease activity and in prolactin levels, and with a significant increase in serum concentrations of L-tyrosine and other CAAs. The increase in free L-tryptophan levels was approaching significance. The findings of this study – improvement in depressive and behavioural symptoms after the start of a gluten-free diet – are supported by the findings of our larger previous retrospective case-control study [7]. Although the results of the amino acid analysis and prolactin levels are only preliminary, they give support to the hypothesis that impaired availability of tryptophan and the possible consequent serotonergic dysfunction may play a role in vulnerability to depressive disorders among adolescents with untreated coeliac disease. A possible role for tyrosine and the brain's catecholamine metabolism (dopamine and noradrenaline) in these disorders cannot, however, be excluded.

The decrease observed in psychiatric symptoms took place regardless of the stress accompanying being diagnosed with a chronic and restrictive illness, and improvement was not explainable in terms of physical symptoms, since both in the present and in our previous study [7], the presence or alleviation of depression showed no association with somatic symptom severity. Our results from adolescents differ from those reported by Addolorato et al. [5]. In their follow-up study on adult patients with coeliac disease, a significant decrease in anxiety symptoms but not in depressive symptoms appeared after one year on a gluten-free diet. Although converging with the findings of Ljungman and Myrdal (20), the few symptoms of our adolescents with coeliac disease adhering to a gluten-free diet in our present and previous [7] studies are thus in contrast to the findings of depressive symptoms [4,5] and disorders [6] as being common among adult patients with coeliac disease, even during diet treatment.

Since the free tryptophan and the tryptophan/CAA ratios in plasma determine the availability of tryptophan to the brain [21], our findings on depressive patients give preliminary support to suggestions of impaired availability of tryptophan as featuring in coeliac-associated depressive and behavioural disorders associated with celiac disease [9,12,13]. As we did not have a control group of healthy adolescents, we cannot say whether L-tryptophan or L-tyrosine levels or both are generally lower among adolescents with coeliac disease, as could be expected based on the findings of Hernanz and Polanco [9], who reported significantly decreased plasma tryptophan and tyrosine concentrations in untreated and treated children with coeliac disease compared to levels in controls.

In the present study, stress-induced biopsy-morning prolactin levels were significantly higher among depressive patients (all girls) and correlated negatively with L-tryptophan/CAA levels. This finding is only preliminary, but it is, however, interesting: Although disturbances in the central serotonergic system have been associated with depressive and impulse-control disorders among adults [see [22]], and children aged 6 to 12 years with a recent suicide attempt have shown lower whole blood tryptophan content [23], serotonergic dysfunction in adolescents with depression is still poorly studied. The prolactin hypersecretion response to the L-5-hydroxytryptophan challenge (L-5HTP) test reported among pre-pubertal girls with major depressive disorder [24] and among healthy children at high risk for major depressive disorder (= high family loading for major depression) [25], may be consistent with dysregulation of the central serotonergic system in childhood major depression [24]. Moreover, alterations in neuroendocrine responses to L-5HTP challenge tests, such as the prolactin hypersecretion and hyposecretion of cortisol found in healthy children, have been suggested to represent a trait marker for depression in children [25]. Thus, the high biopsy morning prolactin levels in depressed adolescents with untreated coeliac disease in the present study could be associated with serotonergic dysfunction. They could also be associated with dopaminergic dysfunction due to impaired availability of tyrosine, since dopamine is known to exert an inhibitory action on prolactin release in the hypothalamus [26]. In the present study, however, pre-diet prolactin levels did not correlate with tyrosine levels. Moreover, the function of the intestinal Catechol-O-Methyl Transferase enzyme (COMT) – known to play an important role in the peripheral O-methylation of catecholamines – remains unstudied in untreated coeliac disease. It is of some theoretical interest that reduced COMT activity in erythrocytes has at least once been associated with conditions such as primary affective disorders in women [27].

On the other hand, in the present study also non-depressed adolescents with coeliac disease had higher than normal biopsy morning prolactin levels. Significantly higher prolactin levels among untreated coeliac children (5–18 years) compared with treated patients has been reported by Reifen et al. [28]. They suggest that prolactin may play a part in the immune modulation of the intestine and could thus serve as a potential marker for coeliac disease activity.

Our preliminary findings on amino acid levels in adolescents with coeliac disease with or without depression are unlikely to be explained by malabsorption, since pre-diet free L-tryptophan and tryptophan ratios were not correlated with the BCAA levels that reflect the level of protein nutrition. It is of theoretical interest that increased production of interferon-γ (IFN-γ), known to be the predominant cytokine produced by gluten-specific T-cells in active coeliac disease [29], can suppress serotonin function both directly and indirectly by enhancing tryptophan and serotonin turnover [30]. Increased IFN-γ [30] and, for instance, such events as a stress-related increase in liver tryptophan pyrrolase enzyme activity [23], may lead to lowered tryptophan levels by the enhanced tryptophan catabolism induced by increased activity of the kynurenine-niacin pathway [30-32], even without malabsorption.

## Conclusion

The alleviation of psychiatric symptoms found among adolescents with coeliac disease after commencement of a gluten-free diet coincides with a rapid decrease in antibody titres indicating coeliac disease activity and in their prolactin levels, and with a significant increase in L-tyrosine and other CAA serum concentrations, and with a nearly significant increase in the free fraction of L-tryptophan. Although these findings are only preliminary, and more research is needed, they give support to previous findings on patients with coeliac disease, suggesting that serotonergic dysfunction due to impaired availability of tryptophan may play a role in vulnerability to depressive and behavioural disorders, also among adolescents with untreated celiac disease. And since diet treatment may alleviate psychiatric symptoms, and earlier diagnosis may have beneficial effects on psychological and even on neurobiological vulnerability to depression, the possibility of psychiatric complications of coeliac disease needs to be taken into account in differential diagnosis of depressive and behavioural disorders.

## Declaration of competing interests

The author(s) declare that they have no competing interests.

## Authors' contributions

PAP, ETI, MAV, ES, VAA contributed to the conception and design of the study, and PAP, MAV, ES to acquisition of the data. All authors (PAP, ETI, MAV, SAK, IS, ES, VAA) contributed to the analysis and interpretation of the data, were involved in drafting and revising the article, and read and approved the final manuscript.

## Pre-publication history

The pre-publication history for this paper can be accessed here:


